# Understanding histone-DNA interactions in the common bean (*Phaseolus vulgaris* L.)

**DOI:** 10.1186/1756-8935-6-S1-P37

**Published:** 2013-03-18

**Authors:** Venu (Kal) Kalavacharla

**Affiliations:** 1 College of Agriculture and Related Sciences, Center for Integrated Biological and Environmental Research (CIBER), Delaware State University Dover, DE 19901, USA

## 

The common bean (*Phaseolus vulgaris* L.) is an important source of plant protein, and member of the legume family, which includes soybean, cowpea, and lentils. With its relatively small genome size, well-characterized cytogenetics, and recently sequenced genome, common bean serves as a good model for legume epigenetics. Accumulation of recent and ongoing bean community-wide genomic and transcriptomic sequence data along with advances in sequencing technology enables the further understanding of the roles of histone-DNA interactions, small RNAs, and cytosine methylation in gene regulation in common bean. Till date, there has not been much progress in studying various epigenomic marks in common bean, and therefore, the long-term objective of this research is to identify protein-DNA interaction sites in the common bean genome to include histones, as well as, non-histone proteins such as transcription factors involved in biotic and abiotic stress. The short-term objective of this work is to develop the first reference epigenomes in four common bean genotypes (G19833, Bat93, Jalo, and Sierra) as related to histone modifications. Common bean genotypes can be broadly classified into those of Andean and Mesoamerican origin, and these genotypes were selected for their importance and use in genomics, as well as breeding. The immediate goal of this work is to identify the hierarchy of binding of histones H4K12_ac_ and H3K9_me2_ in the common bean genome using Illumina sequencing. To achieve this goal, a chromatin immunoprecipitation (ChIP) protocol was developed for common bean, and chromatin from leaves of these four genotypes was isolated and used for ChIP and ChIP-sequencing (ChIP-seq). Sequence analyses of this data is enabling the understanding of the layout of these histone modifications across the genomes of these four genotypes when compared to the reference genome sequence of G19833. This work serves as the first reference histone-DNA interaction map in common bean and lays a foundation for comprehensive analyses and large-scale studies of histone modifications and global protein binding sites in this important plant species, further facilitating the annotation of the common bean genome.

